# Memory CD8^+^ T cell compartment associated with delayed onset of *Plasmodium falciparum* infection and better parasite control in sickle‐cell trait children

**DOI:** 10.1002/cti2.1265

**Published:** 2021-03-19

**Authors:** Claire Loiseau, Boubacar Traore, Aissata Ongoiba, Kassoum Kayentao, Safiatou Doumbo, Didier Doumtabe, Karina P de Sousa, Jamie L Brady, Carla Proietti, Peter D Crompton, Denise L Doolan

**Affiliations:** ^1^ Centre for Molecular Therapeutics Australian Institute of Tropical Health and Medicine James Cook University Cairns QLD Australia; ^2^ Mali International Center of Excellence in Research University of Sciences, Technique and Technology of Bamako Bamako Mali; ^3^ Malaria Infection Biology and Immunity Section Laboratory of Immunogenetics National Institute of Allergy and Infectious Diseases National Institutes of Health Rockville MD USA; ^4^ Present address: School of Life and Medical Sciences Biosciences Research Group University of Hertfordshire Hatfield AL UK

**Keywords:** haemoglobin AS, memory CD8^+^ T cells, *Plasmodium falciparum*, protective immunity, sickle‐cell trait phenotype

## Abstract

**Objectives:**

Study of individuals with protection from *Plasmodium falciparum* (*Pf*) infection and clinical malaria, including individuals affected by the sickle‐cell trait (HbAS), offers the potential to identify cellular targets that could be translated for therapeutic development. We previously reported the first involvement of cellular immunity in HbAS‐associated relative protection and identified a novel subset of memory‐activated NK cells that was enriched in HbAS children and associated with parasite control. We hypothesised that other memory cell subsets might distinguish the baseline profile of HbAS children and children with normal haemoglobin (HbAA).

**Methods:**

Subsets of memory T cells and NK cells were analysed by flow cytometry in paired samples collected from HbAS and HbAA children, at baseline and during the first malaria episode of the ensuing transmission season. Correlations between cell frequencies and features of HbAS‐mediated protection from malaria were determined.

**Results:**

HbAS children displayed significantly higher frequency of memory CD8^+^ T cells at baseline than HbAA children. Baseline frequency of memory CD8^+^ T cells correlated with features of HbAS‐mediated protection from malaria. Exploration of memory CD8^+^ T cell subsets revealed that central memory CD8^+^ T cell frequency was higher in HbAS children than in HbAA children.

**Conclusion:**

This study shows that HbAS children develop a larger memory CD8^+^ T cell compartment than HbAA children, and associates this compartment with better control of subsequent onset of infection and parasite density. Our data suggest that central memory CD8^+^ T cells may play an important role in the relative protection against malaria experienced by HbAS individuals, and further work to investigate this is warranted.

## Graphical abstract

The sickle‐cell trait is associated with protection against malaria, but the role of host immunity has been poorly explored. In this study, we show that central memory CD8^+^ T cells are associated with control of infection and parasite density in sickle‐cell individuals, by analyzing T cell and NK cell subsets in individuals with or without the sickle‐cell trait prior to and at the time of clinical malaria.

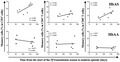

## Introduction

Despite mounting robust innate and adaptive immune responses, humans fail to adequately control *Plasmodium* infection.^1^, ^2^ RTS,S/AS01, also known as Mosquirix^TM^, is the most advanced malaria vaccine candidate.^3^, ^4^ However, multiple studies have demonstrated that its efficacy is suboptimal, too low to achieve malaria eradication, and wanes over time, especially in highly exposed populations.^5^ This lack of an effective long‐lasting vaccine as well as chemoprophylaxis capable of countering multi‐drug resistance demands an increased understanding of key components of the host immune system required for effective and sustained protection. Study of *Plasmodium*‐exposed individuals with relative protection from *Plasmodium falciparum* (*Pf*) infection and clinical malaria^6^ offers the potential to identify cellular and molecular targets that could be translated for the development of successful therapeutics.

The sickle‐cell trait condition, consequence of the heterodimerisation of a normal haemoglobin (Hb) A with an Hb presenting the β6 Glu → Val substitution (HbS), is well known to be associated with a relative protection from *Pf* infection, development of malaria symptoms and severe disease outcomes, but not other infections.^7^, ^8^, ^9^, ^10^, ^11^, ^12^, ^13^, ^14^ A number of molecular mechanisms have been proposed to explain the relative protection observed in HbAS individuals.^15^, ^16^, ^17^, ^18^, ^19^, ^20^, ^21^, ^22^, ^23^, ^24^, ^25^ So far, however, the role of the host immune system in the sickle‐cell trait condition‐associated protection has been poorly explored. Studies focusing on humoral immunity have reported mixed results,^26^, ^27^, ^28^, ^29^, ^30^, ^31^ with the most recent and comprehensive study (protein microarray representing ∼23% of *Pf* proteome) refuting difference in term of *Pf*‐specific antibody responses between HbAS and HbAA children.^31^ Using samples from a cohort of Malian children exposed to intense seasonal *Pf* transmission, we recently published the first study providing evidence that the development of a distinct cellular immune profile may contribute to predispose HbAS children to their relative protection from malaria.^32^ Importantly, we identified a novel subset of memory‐activated NK cells (CD38^dim^CD45RO^+^HLA‐DR^+^ NK cells) and showed that this subset was specifically enriched in HbAS children compared to HbAA children before the start of the *Pf* transmission season and was associated with a higher production of IFN‐γ, a delayed onset of the next malaria episode as well as with a better parasite control during the subsequent malaria episode.^32^ These data highlight the need to identify cell subsets which play a key role in generating an early, rapid, effective and sustained immune response.

In this study, we hypothesised that the cellular memory profile may develop differently in HbAS and HbAA children repeatedly exposed to *Pf*. Therefore, we compared (1) the frequency of seven memory cell subsets (T cell and NK cell subsets) in the PBMC pool, and (2) the frequency of memory cells in those defined immune cell subsets, using paired samples collected prior to the *Pf* transmission season as well as during the first malaria episode of the ensuing transmission season from the same subset of individuals previously studied.^32^ All children experienced at least one episode of clinical malaria. We then investigated potential associations between baseline frequencies of these seven immune cell subsets and four features of HbAS‐mediated protection. Finally, we focused our work on the memory T cell pool and compared profiles of central memory (CM) T cells, effector memory (EM) T cells and effector memory T cells re‐expressing CD45RA (EMRA) in HbAS and HbAA children.

## Results

### Memory CD8^+^ T cells in circulating PBMCs are higher in HbAS individuals than HbAA individuals before the start of the *Pf* transmission season

**Figure 1:**
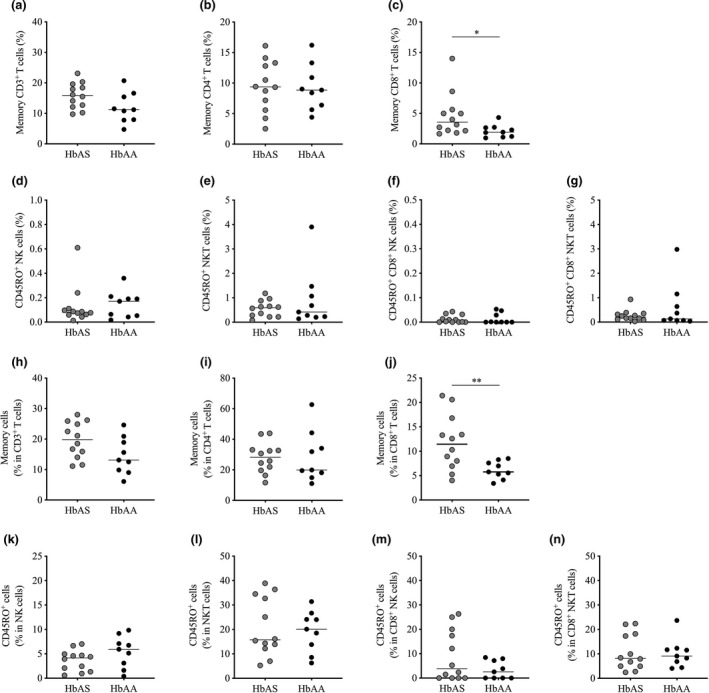
Frequency of circulating memory immune cell subsets in HbAS and HbAA children at baseline. **(a–g)** Frequency of memory cell subsets in the total pool of PBMCs. **(h–n)** Frequency of memory cells in T cell and NK cell subsets. Normality was assessed by the d’Agostino–Pearson normality test. Groups were compared by either an unpaired *t*‐test or the Mann–Whitney *U*‐test depending on the normality. HbAS (*n* = 12) and HbAA (*n* = 9) children. Median bars are shown. *P < 0.05; **P < 0.01.

To investigate whether memory‐activated CD38^dim^ NK cells were the only cell subset differentiating the memory profile of HbAS and HbAA children,^32^ we compared the frequency between HbAS and HbAA children before the start of the *Pf* transmission season (baseline) of (1) the main T cell subsets and (2) NK subsets known to share some functional and phenotypic features with cytotoxic T cells.^34^, ^35^ The blood frequency of memory CD3^+^ T cells (CD45RA^−^CD45RO^+^CD3^+^), memory CD4^+^ T cells (CD45RA^−^CD45RO^+^CD4^+^CD3^+^) and memory CD8^+^ T cells (CD45RA^−^CD45RO^+^CD8^+^CD3^+^), as well as CD45RO^+^ NK cells (CD45RO^+^CD56^+^CD3^−^), CD45RO^+^ NKT cells (CD45RO^+^CD56^+^CD3^+^), CD45RO^+^ CD8^+^ NK cells (CD45RO^+^CD8^+^CD56^+^CD3^−^) and CD45RO^+^ CD8^+^ NKT cells (CD45RO^+^CD8^+^CD56^+^CD3^+^), was determined by flow cytometry (Supplementary figure 1). We first analysed the frequency of each memory cell subset within the total pool of PBMCs (total live single PBMCs). Assessing the whole pool of memory CD3^+^ T cells, we observed a trend towards a lower frequency at baseline in HbAS children than HbAA children (median frequency 15.8% *vs*. 11.2%, respectively, *P* = 0.062, Figure 1a). When considering CD4^+^ and CD8^+^ T cell compartments separately, the frequency of memory CD4^+^ T cells was similar between HbAS and HbAA children (median frequency 9.4% *vs*. 8.9%, *P* = 0.889; Figure 1b), but memory CD8^+^ T cells were significantly more frequent in HbAS children than in HbAA children (median frequency 3.6% *vs*. 1.9%, respectively, *P* = 0.034, Figure 1c). The four subsets of NK cells expressing CD45RO (CD45RO^+^ NK cells; CD45RO^+^ NKT cells; CD45RO^+^ CD8^+^ NK cells; CD45RO^+^ CD8^+^ NKT cells) displayed similar frequency between HbAS and HbAA children (Figure 1d–g). Similarly, memory CD8^+^ T cells were found to be the only cell subset of those evaluated that significantly distinguished HbAS children from HbAA children before the start of the *Pf* transmission season (median frequency 4.98% *vs*. 2.29%, *P* = 0.027; Supplementary figure 2c) when only individuals with paired sample available at the time of first malaria episode of the ensuing *Pf* transmission season were analysed (Supplementary figure 2).

### Memory cells in the circulating CD8^+^ T cell subset are higher in HbAS individuals than HbAA individuals before the start of the *Pf* transmission season

The higher frequency of memory CD8^+^ T cells observed within the total PBMC pool before the start of the *Pf* transmission season in HbAS compared to HbAA children (Figure 1c) could represent either a true modification of the pool of memory cells in CD8^+^ T cells, or a relative redistribution of other populations of PBMCs. Therefore, we next determined the frequency of CD45RO expressing cells within CD3^+^, CD4^+^ and CD8^+^ T cells as well as within NK cell subsets. Consistent with our observations in total PBMCs (Figures 1a and b), there was a trend towards higher frequency of memory cells in CD3^+^ T cells (median frequency 19.8% *vs*. 13.1%, respectively, *P* = 0.064, Figure 1h) and a similar frequency of memory cells in CD4^+^ T cells (median frequency 28.2% *vs*. 19.9%, *P* = 0.937; Figure 1i) in HbAS compared to HbAA. Importantly, however, the frequency of memory cells in CD8^+^ T cells was significantly higher in HbAS children than in HbAA children (median frequency 11.4% *vs*. 5.75%, *P* = 0.010, Figure 1j). There was no apparent difference in the four NK cell subsets (Figure 1k–n). Moreover, the frequencies of memory cells in both CD3^+^ T cells and CD8^+^ T cells were significantly higher in HbAS children than in HbAA children (median frequency 25.9% *vs*. 15.6%, respectively, *P* = 0.030 for CD3^+^ T cells; median frequency 13.4% *vs*. 6.99%, respectively, *P* = 0.004 for CD8^+^ T cells, Supplementary figure 2h, j), when only individuals with paired sample available at the time of first malaria episode of the ensuing *Pf* transmission season were analysed (Supplementary figure 2). Interestingly, this analysis also revealed that HbAS children displayed a significantly higher frequency of memory cells in CD8^+^ NK cells than HbAA children (median frequency 20% *vs*. 3.85%, *P* = 0.003; Supplementary figure 2m).

### Frequency of memory CD8^+^ T cells segregates immune profile of HbAS individuals and HbAA individuals only before the start of the *Pf* transmission season

At the time of first malaria episode of the ensuing *Pf* transmission season, there was no apparent difference in frequency between HbAS and HbAA for any of the seven cell subsets within the total PBMC pool (Supplementary figure 3a–g), or for memory cells within the defined T and NK cell subsets (Supplementary figures 4 and 5a–j), for available paired samples.

Analysis of the cell dynamics between baseline and the first malaria episode for available paired samples revealed a significant difference in frequency only for the CD45RO^+^ NK cell subset within the total PBMC pool in HbAA children (median frequency 0.17% *vs*. 0.03%, respectively, *P* = 0.016; Supplementary figure 3h–n), and for CD45RO^+^ cells in the CD8^+^ NK subset in HbAS children (median frequency 20% *vs*. 5.9%, respectively, *P* = 0.036) (Supplementary figure 5h–n).

### The frequency of memory CD8^+^ T cells before the start of the *Pf* transmission season correlates with features of HbAS‐mediated protection from malaria

**Figure 2:**
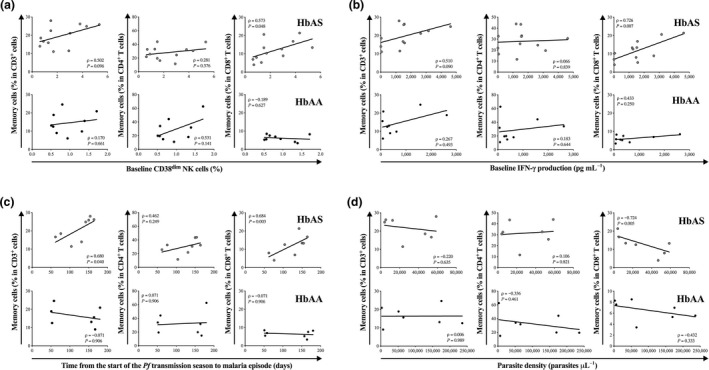
Correlation between the baseline frequency of circulating memory CD3^+^, CD4^+^ and CD8^+^ T cells and features of HbAS‐mediated protection from malaria. Correlation between baseline memory T‐cell frequencies and **(a)** baseline frequency of CD38^dim^ NK cells (HbAS, *n* = 12 and HbAA, *n* = 9), **(b)** baseline production of IFN‐γ production (HbAS, *n* = 12 and HbAA, *n* = 9), **(c)** time from the start of the *Plasmodium falciparum* transmission season to the first malaria episode (HbAS, *n* = 8 and HbAA, *n* = 7), **(d)** parasite density during the first malaria episode (HbAS, *n* = 7 and HbAA, *n* = 7). Pearson’s or Spearman’s correlation coefficients are reported depending on the normality.

We have previously reported that an elevated baseline frequency of memory‐activated CD38^dim^ NK cells within the total PBMC pool may contribute to predispose HbAS children to a relative protection from malaria.^32^ Therefore, we extended our studies to investigate whether the baseline frequency of other defined T and NK cell subsets, and in particular memory CD8^+^ T cells, may correlate with features of HbAS‐mediated protection from malaria: (1) baseline frequency of the protection‐associated CD38^dim^ NK cell subset,^32^ (2) baseline capacity to initiate an IFN‐γ response following mitogen stimulation,^32^ (3) delayed onset of clinical malaria,^13^, ^36^ and (4) capacity to control the parasite density.^8^, ^9^, ^10^ Looking first at the T cell subsets, interestingly, the baseline frequency of memory CD8^+^ T cells was significantly correlated with the baseline frequency of the memory‐activated CD38^dim^ NK cell subset in HbAS children (Figure 2a, top panel), but not in HbAA children (Figure 2a, bottom panel). Moreover, the frequency of memory CD8^+^ T cells determined before the start of the *Pf* transmission season was significantly highly correlated with the capacity to produce an early IFN‐γ response in HbAS children (Figure 2b, top panel), but not in HbAA children (Figure 2b, bottom panel). Importantly, the baseline frequencies of memory CD3^+^ cells and memory CD8^+^ T cells were both significantly correlated with the time from the start of the *Pf* transmission season to malaria episode in HbAS children (Figure 2c, top panel), but not in HbAA children (Figure 2c, bottom panel). Finally, the frequency of memory CD8^+^ T cells before the start of the transmission season was significantly inversely correlated with the parasite density quantified during the first malaria episode of the ensuing transmission season in HbAS children (Figure 2d, top panel), but not in HbAA children (Figure 2d, bottom panel). In both HbAS and HbAA children, the frequency of memory CD4^+^ T cells did not correlate with any of the malaria protection features.

**Figure 3:**
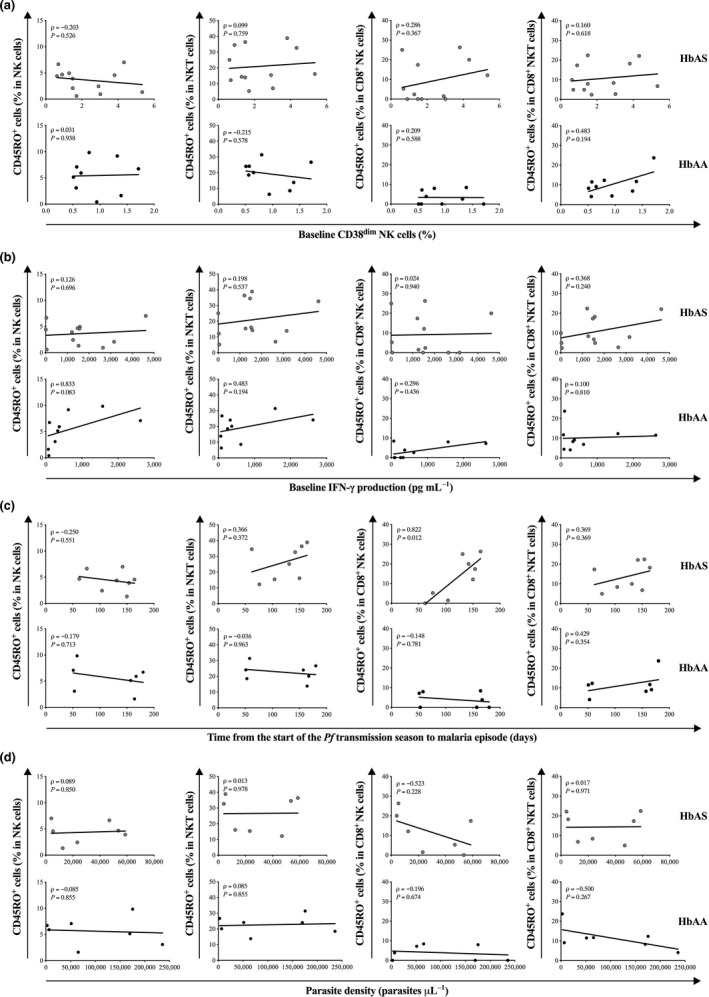
Correlation between the baseline frequency of circulating memory NK cell subsets and features of HbAS‐mediated protection from malaria. Correlation between baseline memory NK cells frequencies and **(a)** baseline frequency of CD38^dim^ NK cells (HbAS, *n* = 12 and HbAA, *n* = 9), **(b)** baseline production of IFN‐γ production (HbAS, *n* = 12 and HbAA, *n* = 9), **(c)** time from the start of the *Plasmodium falciparum* transmission season to the first malaria episode (HbAS, *n* = 8 and HbAA, *n* = 7), **(d)** parasite density during the first malaria episode (HbAS, *n* = 7 and HbAA, *n* = 7). Pearson’s or Spearman’s correlation coefficients are reported depending on the normality.

Looking next at the NK cell subsets (CD45RO^+^ NK cells, CD45RO^+^ NKT cells, CD45RO^+^ CD8^+^ NK cells, CD45RO^+^ CD8^+^ NKT cells) and the four features of HbAS‐mediated protection from malaria (Figure 3a–d), the only significant correlation observed was between the baseline frequency of CD45RO^+^ CD8^+^ NK cells and the time from the start of the *Pf* transmission season to malaria episode in HbAS children (Figure 3c, top panel).

These data support the concept that an abundant pre‐existing memory CD8^+^ T cell compartment may contribute to the relative malaria protection observed in HbAS children.

### Higher frequency of memory CD8^+^ T cells in HbAS individuals before the start of the *Pf* transmission season is driven by central memory T cells

**Figure 4:**
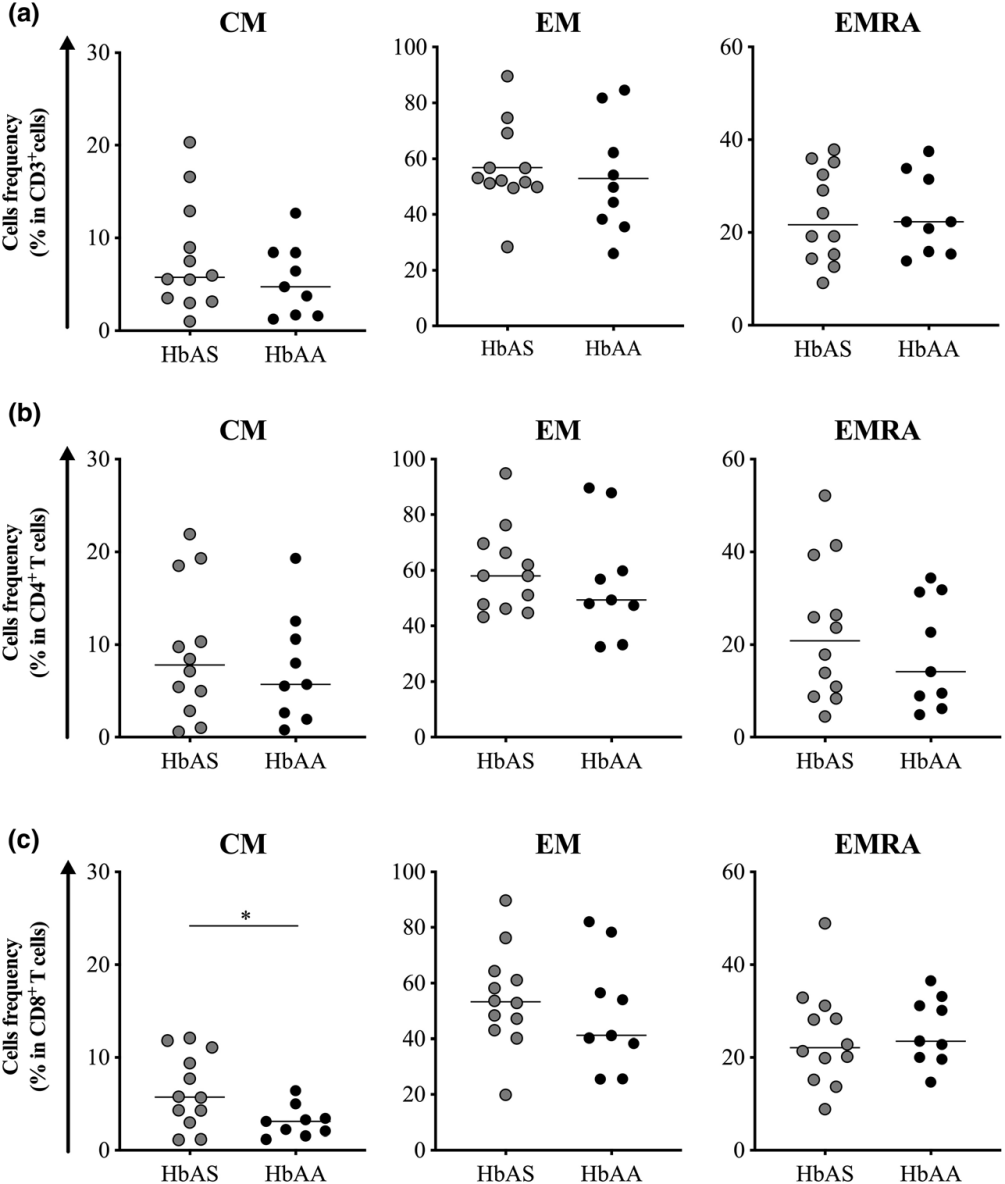
Frequency of circulating memory T cell subsets in HbAS and HbAA children at baseline. Frequency of CM, EM and EMRA cells in **(a)** CD3^+^ T cells, **(b)** CD4^+^ T cells and **(c)** CD8^+^ T cells. Normality was assessed by the d’Agostino–Pearson normality test. Groups were compared by an unpaired *t‐*test. HbAS children (*n* = 12) and HbAA children (*n* = 9). Median bars are shown. *P < 0.05.

The memory T cell pool has traditionally been divided into two major subsets based on the expression of the receptor CCR7^37^: central memory T cells (CM: CCR7^+^CD45RO^+^CD45RA^−^) and effector memory T cells (EM: CCR7^−^CD45RO^+^CD45RA^−^). More recently, a third population of effector memory T cells re‐expressing CD45RA (EMRA: CCR7^−^CD45RO^−^CD45RA^+^) has been described.^38^ Therefore, we compared the frequencies of these three subsets of memory cells in the pool of CD3^+^ cells, CD4^+^ T cells and CD8^+^ T cells between HbAS and HbAA children. Baseline frequencies of CM, EM and EMRA in both the pool of CD3^+^ cells and CD4^+^ T cells were similar between HbAS and HbAA children (Figure 4a and b). However, the baseline frequency of CM in CD8^+^ T cells was significantly higher in HbAS children than HbAA children (median frequency 5.7% *vs*. 3.1%, *P* = 0.030, Figure 4c, left panel); EM and EMRA frequencies in CD8^+^ T cells were similar (median frequency 53.2% *vs*. 41.2%, *P* = 0.521 for EM; 22.1% *vs*. 23.5%, *P* = 0.731 for EMRA cells, Figure 4c). Similarly, the baseline frequency of CM in CD8^+^ T cells was significantly higher in HbAS children than HbAA children (median frequency 7.7% *vs*. 3.3%, *P* = 0.020, Supplementary figure 6c, left panel) when only individuals with paired sample available at the time of first malaria episode of the ensuing *Pf* transmission season were analysed (Supplementary figure 6).

**Figure 5:**
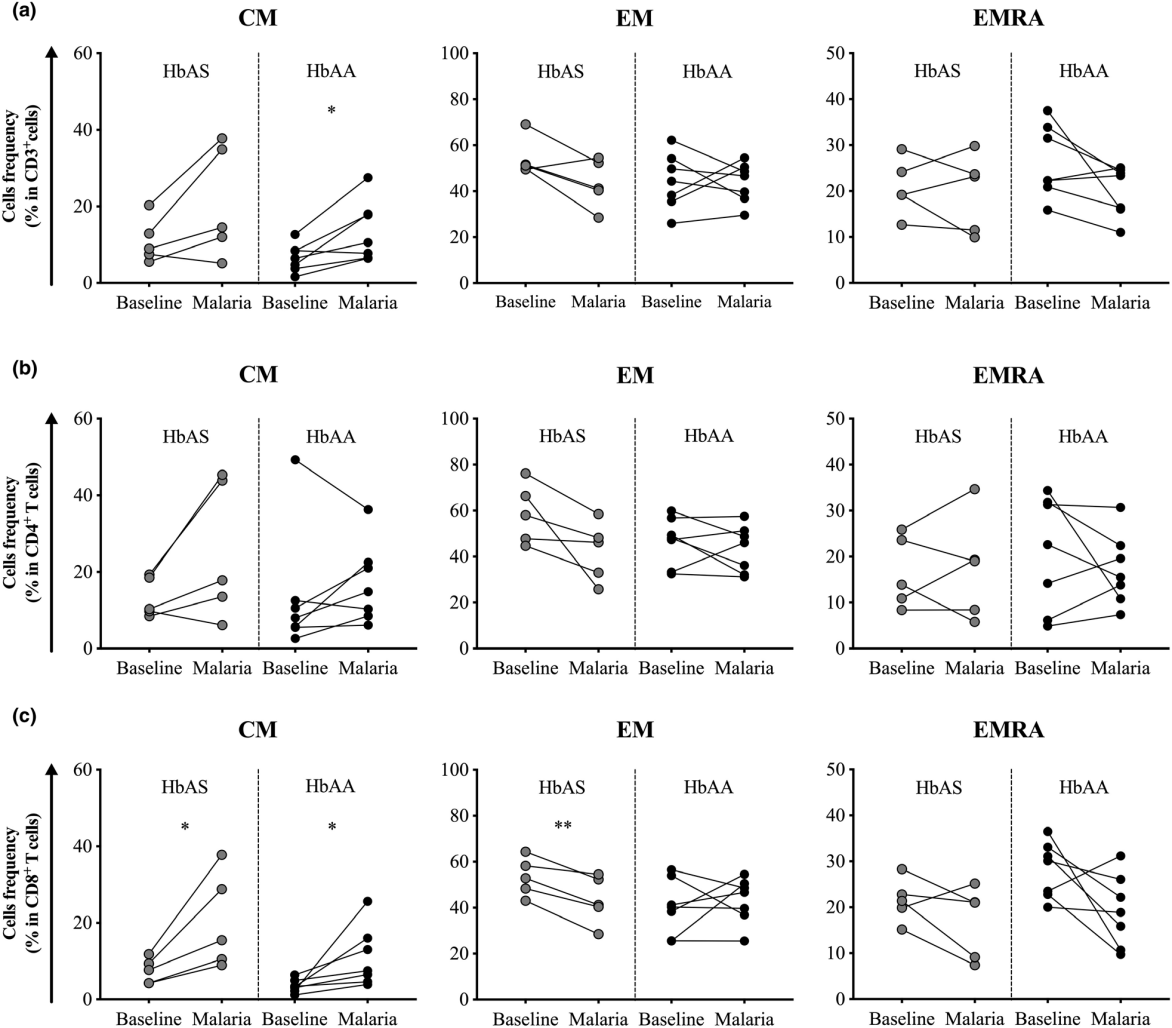
Dynamic of circulating memory T cell subsets in HbAS and HbAA children between baseline and the first malaria episode of the following *Plasmodium falciparum* (*Pf*) transmission season. Dynamic of CM, EM and EMRA cells in **(a)** CD3^+^ T cells, **(b)** CD4^+^ T cells and **(c)** CD8^+^ T cells between baseline and the first malaria episode of the following *Pf* transmission season Normality was assessed by the Shapiro–Wilk normality test. Groups were compared by either the paired *t*‐test or Wilcoxon’s matched‐pairs signed rank test depending on the normality. HbAS children (*n* = 5) and HbAA children (*n* = 7). Median bars are shown. *P < 0.05; **P < 0.01.

Finally, analysis at the time of the first malaria episode of the ensuing *Pf* transmission season (Supplementary figure 7) did not reveal any difference between HbAA and HbAS children. Of importance, the analysis of cell dynamics revealed that the frequency of CM in CD8^+^ T cells significantly increased in both HbAS and HbAA children (median frequency 7.7% *vs*. 15.5%, *P* = 0.038 for HbAS children; 3.3% *vs*. 7.5%, *P* = 0.047, Figure 5 for HbAA children). In contrast, the frequency of EM in CD8^+^ T cells significantly decreased in HbAS (median frequency 52.90% baseline *vs*. 41.20% malaria, *P* = 0.006; Figure 5), consistent with migration of EM CD8^+^ T cells from the peripheral circulation to the infected tissue (i.e. liver).

## Discussion

Herein, we report for the first time the existence of a memory CD8^+^ T cell compartment abundant in HbAS children compared to HbAA children before the start of the *Pf* transmission season, providing further support for a distinct cellular immune response between HbAS and HbAA individuals in the context of *Pf* infection.^32^ Importantly, we show that the baseline frequency of the memory CD8^+^ T cell compartment strongly correlates with features of HbAS‐mediated protection from malaria, and identify central memory CD8^+^ T cells as the important memory subset.

Thus far, development of cellular immunity in the context of *Pf* infection and identification of key cell subsets associated with relative protection from both infection and disease development have been poorly investigated in HbAS individuals. Despite the fact that CD8^+^ T cells are known to play a major role in the host immune response to early stages of *Pf* infection,^39^ to our knowledge this T cell subset has not been explored in HbAS individuals repeatedly exposed to *Pf*. Our work establishes for the first time that (1) the memory CD8^+^ T cell compartment is significantly more abundant in HbAS children than in HbAA children despite presumably similar parasite exposure; and (2) the baseline frequency of memory CD8^+^ T cells significantly correlates with features of protection in HbAS, including the baseline capacity to initiate an IFN‐γ response, delayed onset of malaria and the capacity to control the parasite density. We are not aware of any studies looking at memory cells in HbAS individuals in non‐malaria endemic regions, but as individuals in those regions would not experience clinical malaria, any such study would be limited (e.g. to assessment of the longevity of immunity) and thus would not directly relate to the findings reported herein.

Although memory T cells are often considered as a bulk population of cells, expression of CCR7 distingishes CM and EM T cells.^37^ CM T cells continuously patrol between the blood and the T cell zone of secondary lymphoid tissues scanning for the presence of pathogenic motifs presented by antigen‐presenting cells, when re‐stimulated CM T cells then undergo a massive proliferation, differentiate into effector cells, and migrate to infected peripheral tissue.^40^, ^41^ By contrast, EM recirculate between the blood and peripheral tissue, and following pathogen re‐exposure, EM cells rapidly provide infected peripheral tissue with their effector functions.^40^, ^41^, ^42^ Herein, we demonstrate that the pool of CM CD8^+^ T cells before the start of the *Pf* transmission season was significantly higher in HbAS children than in HbAA children. This observation suggests that HbAS individuals may display a higher potential to scan for the presence of pathogens, providing a quick and early immune response and therefore supporting a better parasite control. In accordance with this hypothesis, our analysis of the cell dynamics between baseline and the first malaria episode of the following transmission season in HbAS showed a significant expansion of CM CD8^+^ T cells. This analysis also revealed a significantly reduced blood frequency of EM CD8^+^ T cells in HbAS children between baseline and the first malaria episode, suggesting that early on, in HbAS children, EM CD8^+^ T cells might quickly migrate to the infected tissue (i.e. liver) to help in the early control of parasite replication.

It should be noted, however, that samples sizes studied herein are insufficient to allow for definitive conclusions. Also, although our HbAS and HbAA individuals were similar in terms of age, sex, body weight, area of residence, haemoglobin concentration and prior malaria exposure, we cannot rule out unknown potential confounders unrelated to malaria that might impact the observed outcomes, such as other genetic anomalies, HIV status or other infections. Further work, preferably in other populations, is warranted.

Our work demonstrates the importance of studying the cellular immune response in human populations naturally displaying relative protection from *Pf* infection and clinical malaria to better understand immunity to malaria and facilitate the development of effective therapeutic interventions. Abundance of the CD8^+^ T cell memory compartment and its correlation with features of HbAS‐associated relative protection from infection and disease protection suggest that induction of a memory CD8^+^ T cell response (as well as memory NK response^32^) should be considered in the design of new vaccine candidates against *Plasmodium* infection.

## Methods

### Study participants

Subjects included in this study were a subset from 695 individuals aged 3 months to 25 years, enrolled in a large cohort study conducted in 2011 in Kalifabougou, Mali, where intense seasonal *Pf* transmission occurs during a predictable 6‐month period from July through December, and where repeated infections are common.^33^ At baseline (before the start of the *Pf* transmission season, in May), all subjects displayed a haemoglobin level > 7 g dL^−1^, an axillary temperature < 37.5°C, and were free of acute systemic illness and chronic disease. Malaria episodes were detected by passive (self‐reporting to local health centre) and active (medical examination and PCR, every 2 weeks from enrolment through December) surveillance. Malaria episodes were defined as parasitemia ≥ 2500 parasites μL^−1^, an axillary temperature ≥ 37.5°C and/or signs and symptoms consistent with malaria.^33^ We focused on individuals aged 7–13 years, which is the age range during which the acquisition of clinical immunity to uncomplicated malaria begins in areas of intense malaria transmission. From this subset, we randomly selected nine HbAA and 12 HbAS children free of *Pf* infection (determined by PCR), with blood samples and clinical data available before the start of the *Pf* transmission season (Table 1).^32^ There was no difference between HbAA and HbAS children for time to first clinical episode or first infection (*P* = 0.4798, Table 1; Supplementary figure 8). At the first malaria episode of the ensuing transmission season, paired clinical data were available for seven HbAA and eight HbAS individuals (Table 1) and paired blood samples were available for seven HbAA and five HbAS individuals. The groups were similar in terms of sex ratio (*P* = 0.447), body weight (*P* = 0.284), age (*P* = 0.477) and haemoglobin concentration (*P* = 0.955) (Table 1). During the first malaria episode, the axillary temperature was significantly increased in HbAA children (*P* = 0.012; Table 1) and HbAS children (*P* = 0.007; Table 1) compared to baseline; a trend towards a better parasite control was observed in HbAS children compared to HbAA children (*P* = 0.086; Table 1).

**Table 1:** Clinical characteristics

	Baseline		Malaria episode		*P*‐value
	HbAA	HbAS	HbAA	HbAS	
	(*n* = 9)	(*n* = 12)	(*n* = 7)	(*n* = 8)	
Sex ratio (F/M)	1.25	0.50	1.33	0.33	NS^b^
Body weight (kg)^a^	19.0 [17–27]	23.0 [19–34]	21.0 [17–29]	24.0 [19–37]	NS^c^
Axillary temperature (ºC)^a^	36.4 [35.9–36.8]	36.5 [36.0–37.1]	38.4 [36.0–39.8]	37.8 [37.0–39.4]	HbAA *P* < 0.05^d^ HbAS *P* < 0.01^d^
Age (years)^a^	8 [7–11]	9 [7–13]	8.0 [7–11]	8.5 [7–13]	NS^c^
Haemoglobin concentration (g dL^−1^)^a^	11.5 [10.1–13.9]	11.5 [10.6–13.9]	12.0 [9.6–14.6]	11.9 [10.0–13.4]	NS^e^
Parasite density (parasites μL^−1^)^a^			65 400 [2725–235 725]	23 250* [4050–58 700]	NS^f^

F, female; Hb, Haemoglobin; M, male; NS, not significant.

^a^ Median values are reported, and the range is indicated in square brackets.

^b^ Chi‐square test.

^c^ Mann‐Whitney *U*‐test.

^d^ Paired *t*‐test.

^e^ One‐way ANOVA.

^f^ Unpaired *t*‐test with Welch’s correction.

^*^ qPCR data for parasite density are missing for one HbAS individual.

#### Samples

Venous blood (8 mL) was collected before the start of the *Pf* transmission season and at the time of the first malaria episode into sodium heparin tubes (BD Vacutainer CPT, Franklin Lakes, NJ) and processed in the local laboratory within 3 h of collection. PBMCs were isolated by standard density gradient centrifugation and stored at −80°C overnight before long‐term storage in liquid nitrogen.

#### Haemoglobin type

Haemoglobin typing for HbA, HbS and HbC was determined by high‐performance liquid chromatography using a D‐10 haemoglobin analyser (Bio‐Rad, Hercules, CA, USA)^32^.

#### Parasite density

Parasite density was quantified from genomic DNA of *Pf* asexual parasites extracted from dried blood spots as reported previously^32^. Briefly, the *Pf* 18S rRNA gene was amplified by 15‐cycle standard PCR amplification followed by qPCR. The limit of detection of the nested qPCR was ~ 0.5 parasites μL^−1^.

#### Immunophenotyping of peripheral blood lymphocytes

PBMCs isolated from peripheral blood samples from HbAS and HbAA children were stimulated with 20 ng mL^−1^ of PMA and 1000 ng mL^−1^ of Ionomycin (Sigma‐Aldrich, St. Louis, MO) for 6 hrs at 37°C. Cells were then stained for 20 min with two panels of anti‐human monoclonal antibodies (Supplementary table 1) for the main T cell and NK cell subsets, including: memory CD3^+^ T cells (CD45RA^−^CD45RO^+^CD3^+^), memory CD4^+^ T cells (CD45RA^−^CD45RO^+^CD4^+^CD3^+^), memory CD8^+^ T cells (CD45RA^−^CD45RO^+^CD8^+^CD3^+^), CD45RO^+^ NK cells (CD45RO^+^CD56^+^CD3^−^), CD45RO^+^ NKT cells (CD45RO^+^CD56^+^CD3^+^), CD45RO^+^ CD8^+^ NK cells (CD45RO^+^CD8^+^CD56^+^CD3^−^) and CD45RO^+^ CD8^+^ NKT cells (CD45RO^+^CD8^+^CD56^+^CD3^+^). Samples were analysed using a BD LSRFortessaX20 driven by FACSDiva software (BD Biosciences, Franklin Lakes, NJ, USA). FlowJo software version 10.4 (Ashland, OR, USA) was used for gating.

#### CD38^dim^ NK cells frequency

The frequency of CD38^dim^ NK cells was determined as previously described.^32^ Briefly, anti‐human CD3‐APC‐H7 (SK7), CD56‐PE (B159) (both from BD Biosciences) and anti‐human CD38‐BV650 (HB‐7; BioLegend, San Diego, CA, USA) monoclonal antibodies were used to characterise CD38^dim^ NK cells. Analysis was performed using a BD LSRFortessaX20 driven by FACSDiva software (BD Biosciences). FlowJo software version 10.4 was used for gating.

#### IFN‐γ quantification

IFN‐γ concentration was quantified as previously described.^32^ Briefly, IFN‐γ concentration was quantified in the supernatant of stimulated PBMCs using the Human ProcartaPlex Panel (Invitrogen, Carlsbad, CA) following the manufacturer’s instructions. Detection was performed using a MagPIX analyzer driven by Xponent software (Luminex Corporation, Austin, TX, USA).

#### Statistical analysis

Statistical analyses were performed with Prism 7 (GraphPad Software, Inc., San Diego, CA, USA). Statistical tests used are indicated for each table and figure. Normality was assessed by either the d’Agostino‐Pearson normality test or Shapiro‐Wilk normality test, if ‘*n*’ was too small. *P*‐values < 0.05 were considered statistically significant.

#### Study approval

The Malian cohort study was conducted in compliance with all applicable federal regulations governing protection of human subjects and was approved by the Ethics Committee ((FWA #00001769) of the Faculty of Medicine, Pharmacy and Dentistry at the University of Sciences, Techniques and Technologies of Bamako (#2011/37/FMPOS and 2018/181/FMPOS) and the Institutional Review Board of the National Institute of Allergy and Infectious Diseases, National Institutes of Health (#07‐I‐N141, #11‐I‐N126 and OHSRP #12362). The study was registered on http://www.clinicaltrials.gov (NCT01322581). Written informed consent was obtained from the parents or guardians of participating children. The laboratory study with samples collected during that study was approved by the Queensland Institute of Medical Research Human Research Ethics Committee (#P1111) and James Cook University Human Research Ethics Committee (#H7735).

## Conflict of interest

The authors declare no conflict of interest.

## Author contributions

**Claire Loiseau:** Conceptualization; Data curation; Formal analysis; Investigation; Methodology; Writing‐original draft; Writing‐review & editing. **Boubacar Traore:** Resources. **Aissata Ongoiba:** Investigation; Resources. **Kassoum Kayentao :** Investigation; Resources. **Safiatou Doumbo :** Investigation; Resources. **Didier Doumtabe:** Project administration; Resources. **Karina de Sousa:** Project administration. **Jamie Brady:** Project administration. **Carla Proietti:** Project administration; Resources. **Peter Crompton:** Funding acquisition; Resources; Writing‐review & editing. **Denise Doolan:** Conceptualization; Funding acquisition; Investigation; Project administration; Supervision; Writing‐original draft; Writing‐review & editing.
