# Supplementary material for: Memory CD8+ T cell compartment associated with delayed onset of Plasmodium falciparum infection and better parasite control in sickle‐cell trait children
Source: Clin Transl Immunology. 2021 Mar 19;10(3):e1265. doi: 10.1002/cti2.1265 (PMC7979311; doi:10.1002/cti2.1265)
Supplement: Supplementary file 1 [file CTI2-10-e1265-s001.pdf]

Supplementary Table 1: Flow cytometry staining panels.

Marker	Fluorochrome	Clone	Company	Catalog number
**Memory T cell-panel**				
CD3	APC-H7	SK7	BD Pharmingen	560176
CD4	BUV495	SK3	BD Horizon	562652
CD8	PE-Cy7	SK1	BioLegend	344712
CD45RA	FITC	HI100	BD Pharmingen	555488
CD45RO	BV510	UCHL1	BioLegend	304246
CD197 (CCR7)	AF647	150503	BD Pharmingen	560816
**Memory NK cell-panel**				
CD3	APC-H7	SK7	BD Pharmingen	560176
CD8	PE-Cy7	SK1	BioLegend	344712
CD56	PE	B159	BD Pharmingen	555516
CD45RO	BV510	UCHL1	BioLegend	304246

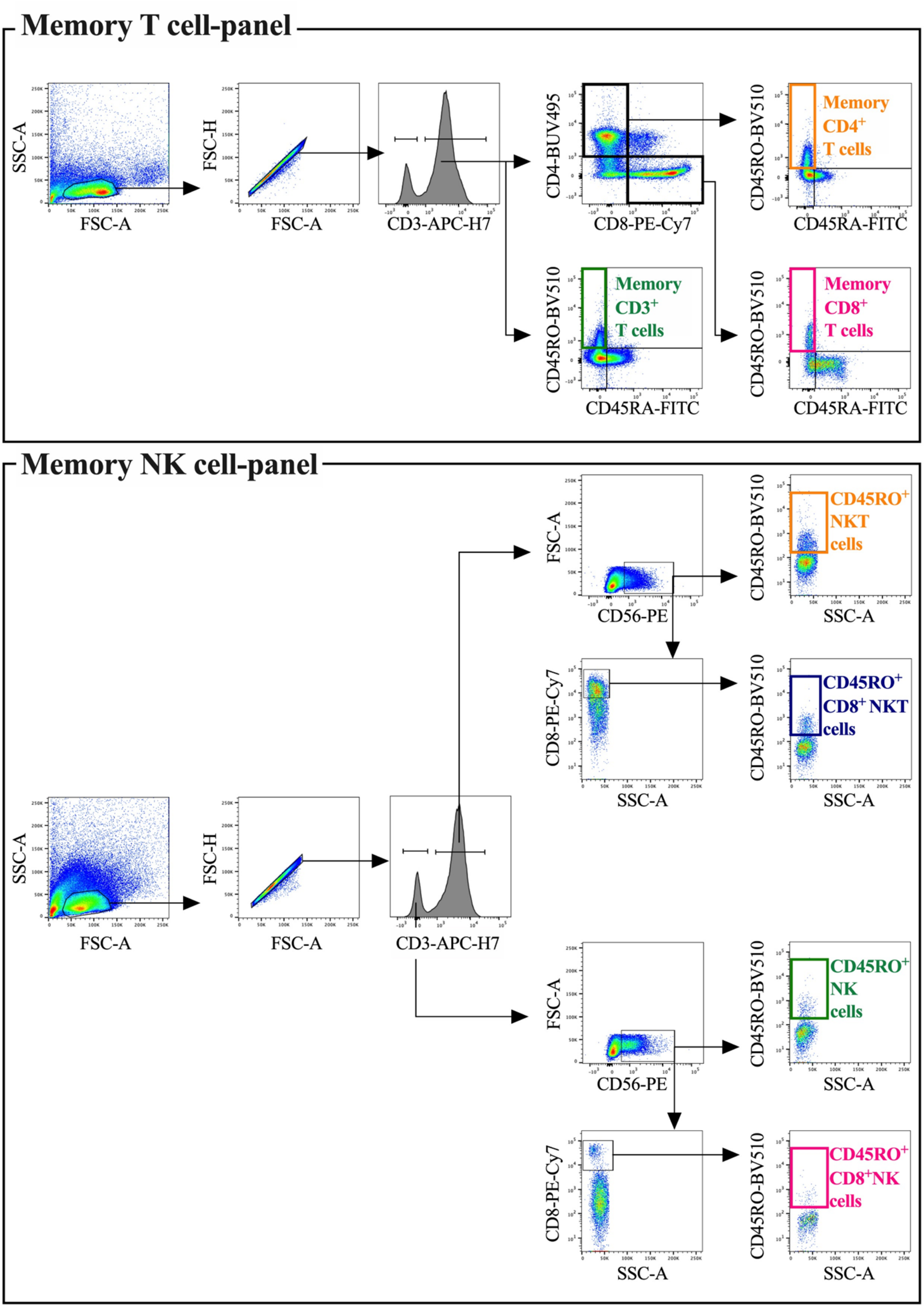

Supplementary figure 1. Flow cytometry gating strategy for memory T and NK cell subsets. Flow cytometry analysis of blood frequency of memory $CD3^+$ T cells ($CD45RA^-CD45RO^+CD3^+$), memory $CD4^+$ T cells ($CD45RA^-CD45RO^+CD4^+CD3^+$), and memory $CD8^+$ T cells ($CD45RA^-CD45RO^+CD8^+CD3^+$); as well as $CD45RO^+$ NK cells ($CD45RO^+CD56^+CD3^-$), $CD45RO^+$ NKT cells ($CD45RO^+CD56^+CD3^+$), $CD45RO^+$ $CD8^+$ NK cells ($CD45RO^+CD8^+CD56^+CD3^-$) and $CD45RO^+$ $CD8^+$ NKT cells ($CD45RO^+CD8^+CD56^+CD3^+$), from one representative experiment.

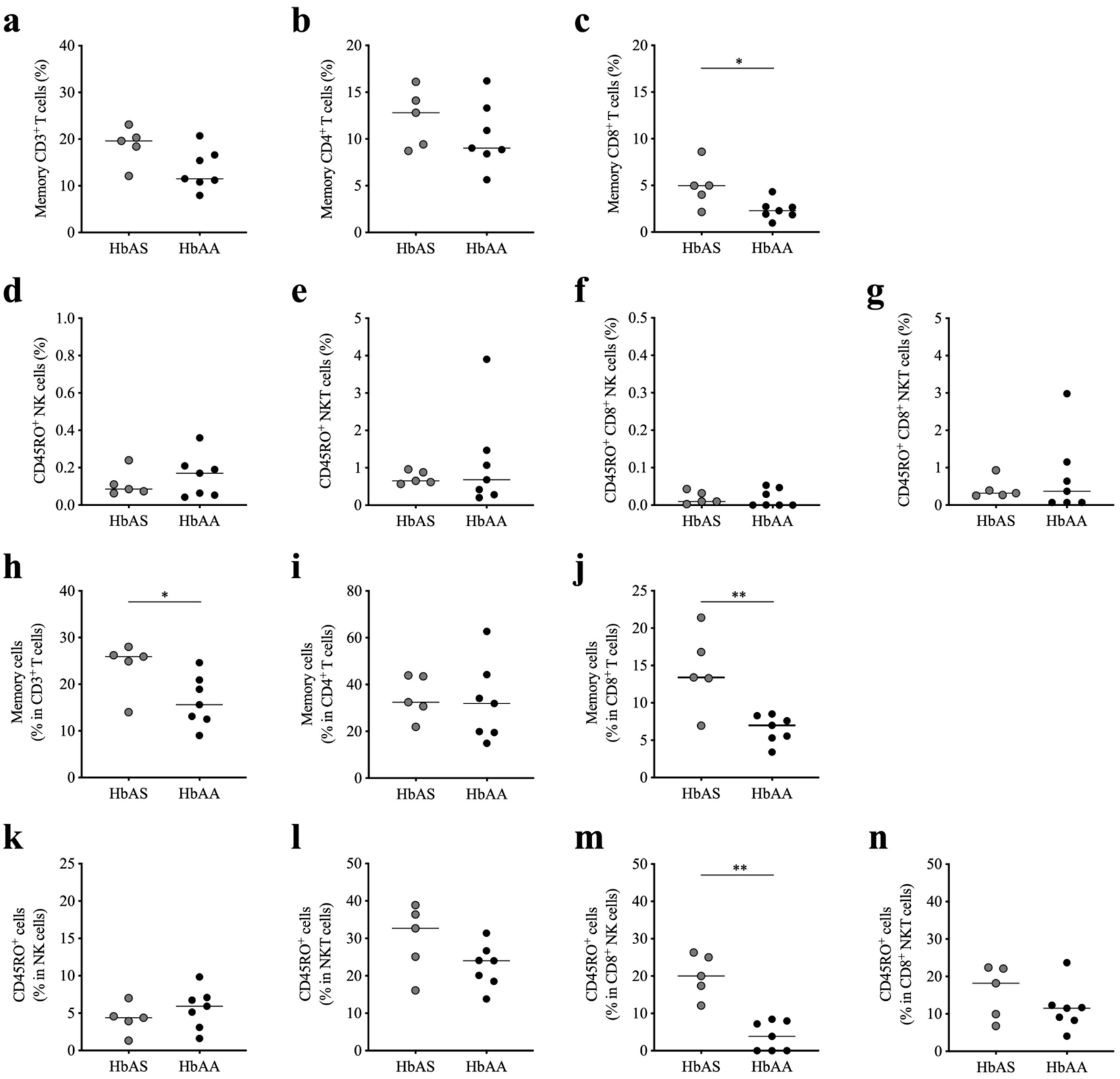

Supplementary figure 2. Frequency of circulating memory immune cell subsets in HbAS and HbAA children at baseline.(**a-g**) Frequency of memory cell subsets in the total pool of PBMCs. (**h-n**) Frequency of memory cells in T and NK cell subsets. Normality was assessed by the Shapiro-Wilk normality test. Groups were compared by either an unpaired *t*-test or the Mann–Whitney U-test depending on the normality. Only individuals with paired sample available at the time of first malaria episode of the ensuing *Pf* transmission season were analysed; HbAS (n = 5) and HbAA (n = 7) children. Median bars are shown. $^{*}P < 0.05$; $^{**}P < 0.01$.

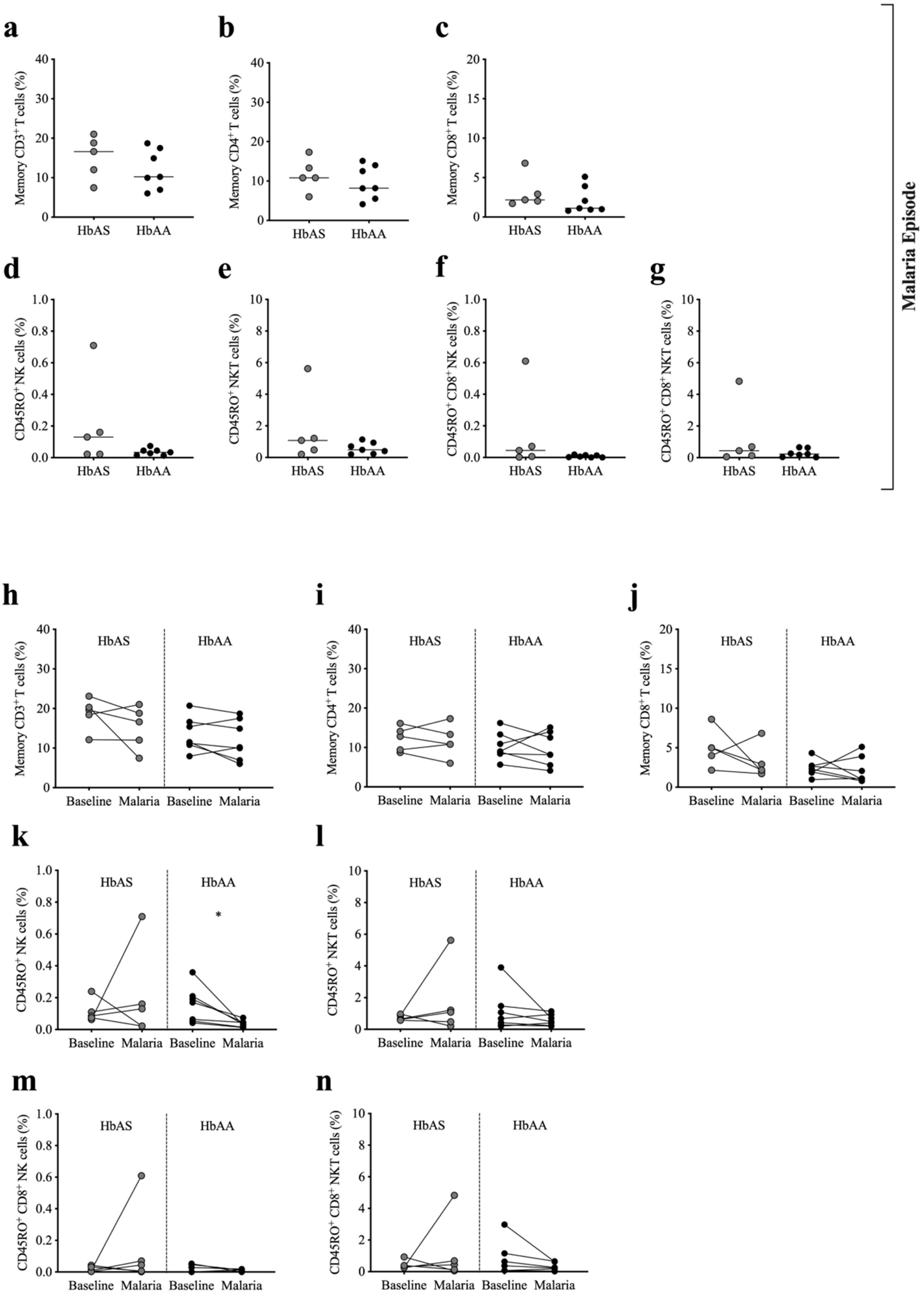

Supplementary figure 3. Memory cell subsets in the total pool of PBMCs in HbAS and HbAA children during the first malaria episode. (**a-g**) Frequency of memory cell subsets in the total pool of PBMCs. Normality was assessed by the Shapiro-Wilk normality test. Groups were compared by either an unpaired *t*-test or the Mann–Whitney U-test depending on the normality. Median bars are shown. (**h-n**) Cell dynamic between baseline and the first malaria episode of the following *Pf* transmission season. Normality was assessed by the Shapiro-Wilk normality test. Groups were compared by either paired *t*-test or Wilcoxon's matched-pairs signed rank test depending on the normality. $^{*}P < 0.05$. HbAS children (n = 5) and HbAA children (n = 7).

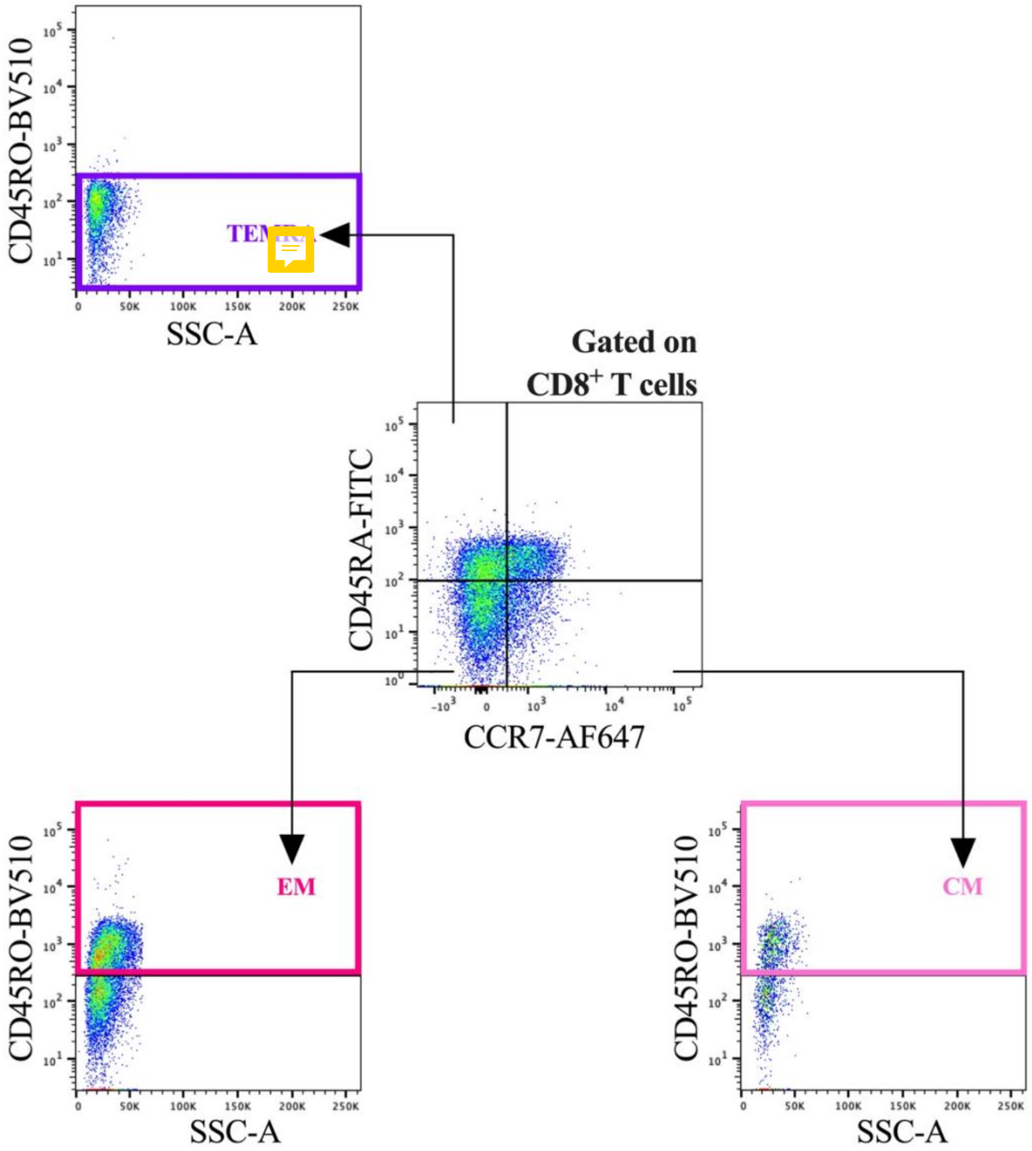

Supplementary figure 4. Flow cytometry gating strategy for memory T cell subsets: CM, EM and TEMRA. Flow cytometry analysis of central memory T cells (CM: $CCR7^{+}CD45RO^{+}CD45RA^{-}$), effector memory T cells (EM: $CCR7^{-}CD45RO^{+}CD45RA^{-}$), and effector memory T cells re-expressing CD45RA (TEMRA: $CCR7^{-}CD45RO^{-}CD45RA^{+}$), from one representative experiment.

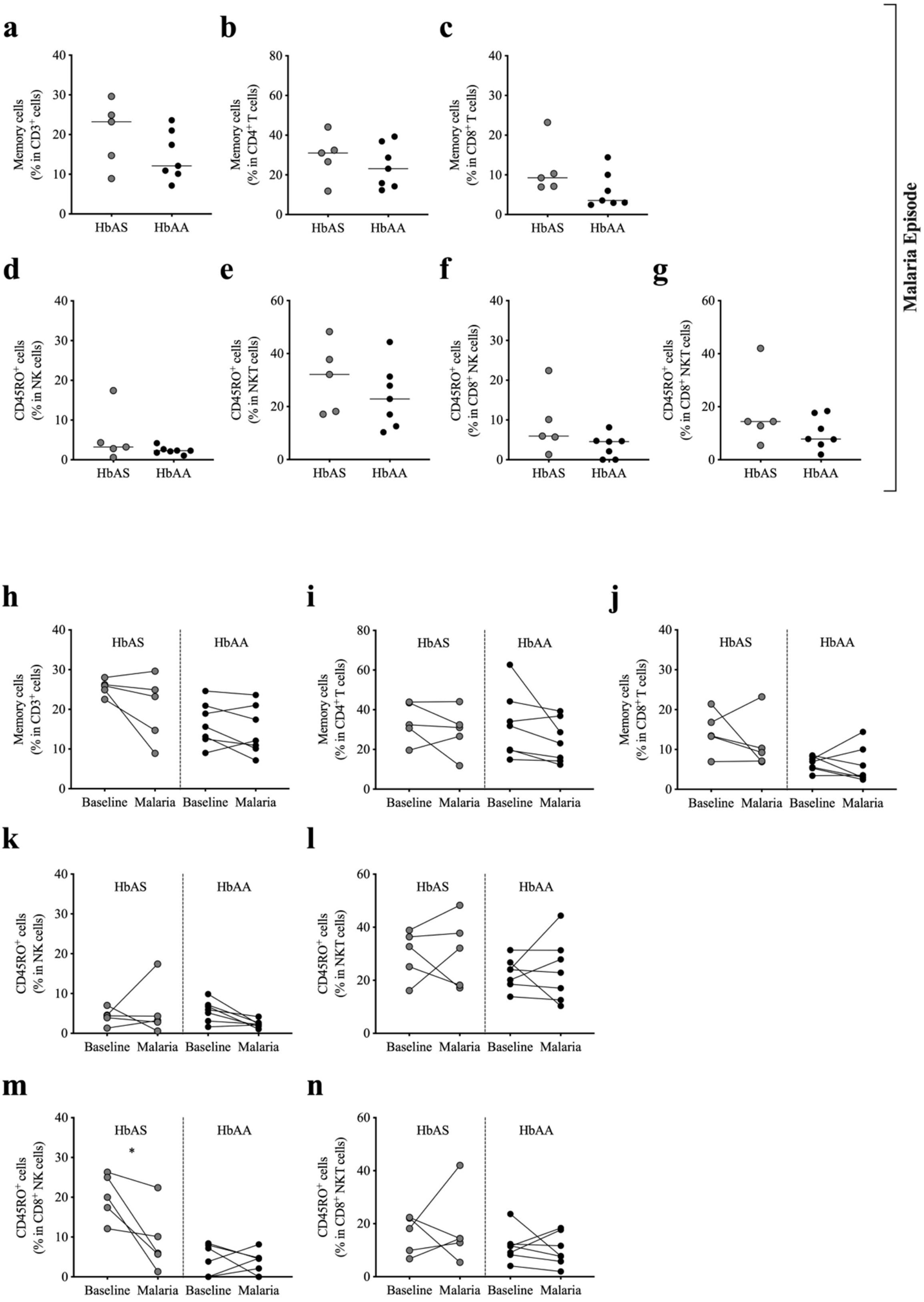

Supplementary figure 5. Memory cells in T and NK cell subsets in HbAS and HbAA children during the first malaria episode. (**a-g**) Frequency of memory cells in T and NK cell subsets. Normality was assessed by the Shapiro-Wilk normality test normality test. Groups were compared by either an unpaired *t*-test or the Mann–Whitney U-test depending on the normality. Median bars are shown. (**h-n**) Cell dynamic between baseline and the first malaria episode of the following *Pf* transmission season. Normality was assessed by the Shapiro-Wilk normality test. Groups were compared by either paired *t*-test or Wilcoxon's matched-pairs signed rank test depending on the normality. *$P < 0.05$. HbAS children (n = 5) and HbAA children (n = 7).

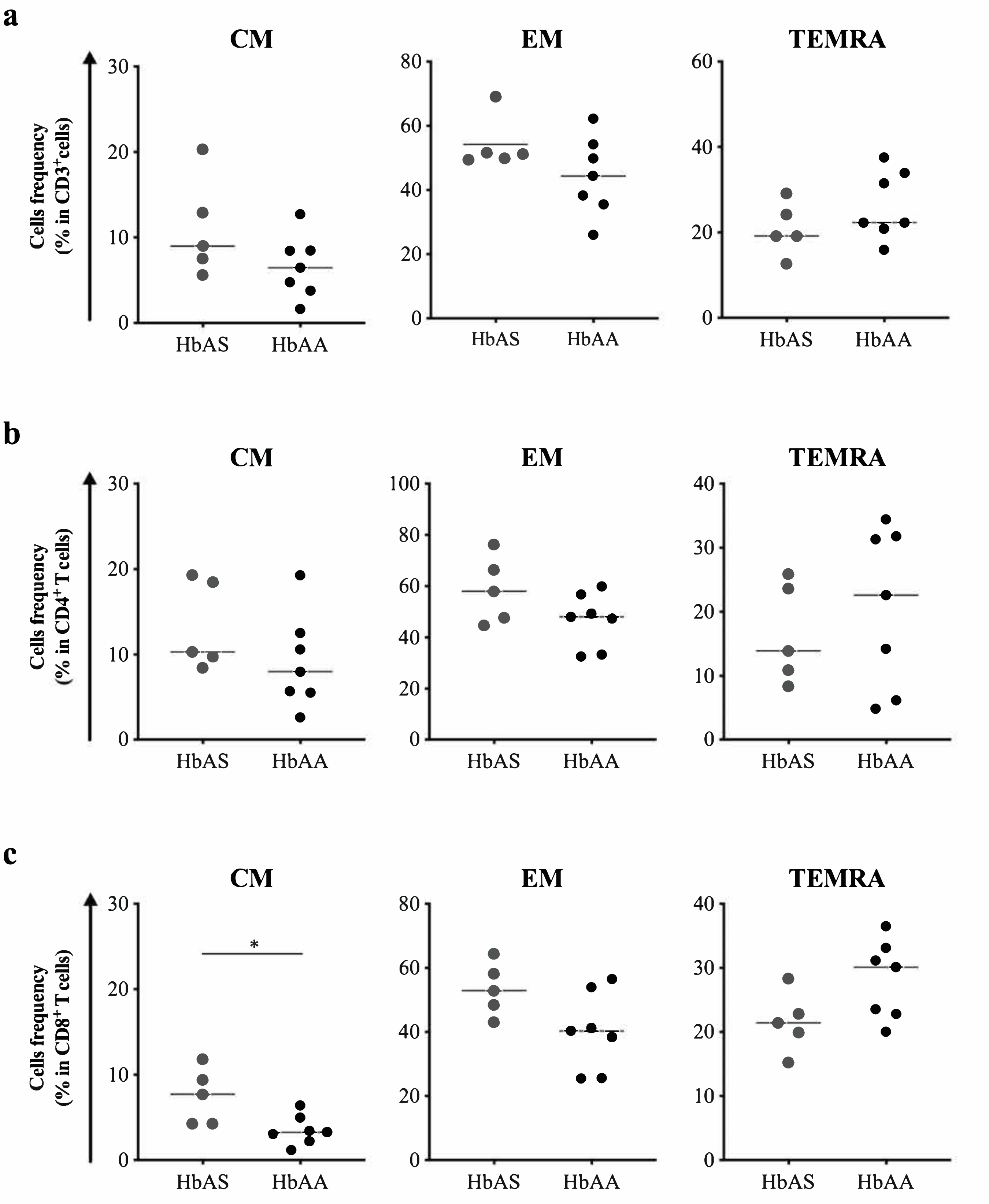

Supplementary figure 6. Frequency of circulating memory T cell subsets in HbAS and HbAA children at baseline. Frequency of CM, EM and TEMRA cells in (**a**) $CD3^+$ T cells, (**b**) $CD4^+$ T cells and (**c**) $CD8^+$ T cells. Normality was assessed by the Shapiro-Wilk normality test. Groups were compared by unpaired *t*-test. HbAS children (n = 5) and HbAA children (n = 7). Median bars are shown. *$P < 0.05$.

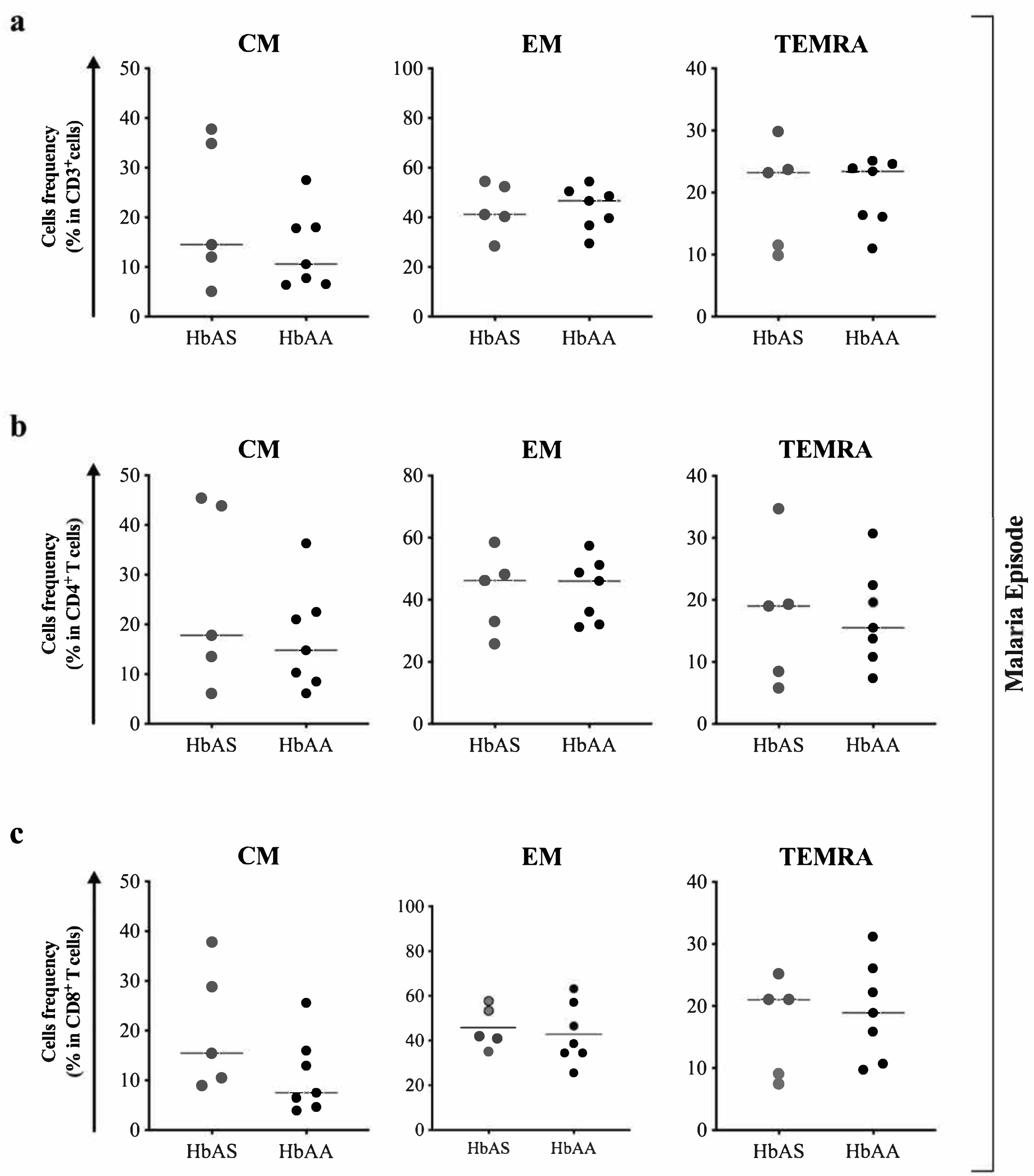

Supplementary figure 7. Frequency of circulating memory T cell subsets in HbAS and HbAA children during the first malaria episode. Frequency of CM, EM and TEMRA cells in (**a**) $CD3^+$ T cells, (**b**) $CD4^+$ T cells and (**c**) $CD8^+$ T cells. Normality was assessed by the Shapiro-Wilk normality test. Groups were compared by unpaired *t*-test. HbAS children (n = 5) and HbAA children (n = 7). Median bars are shown.

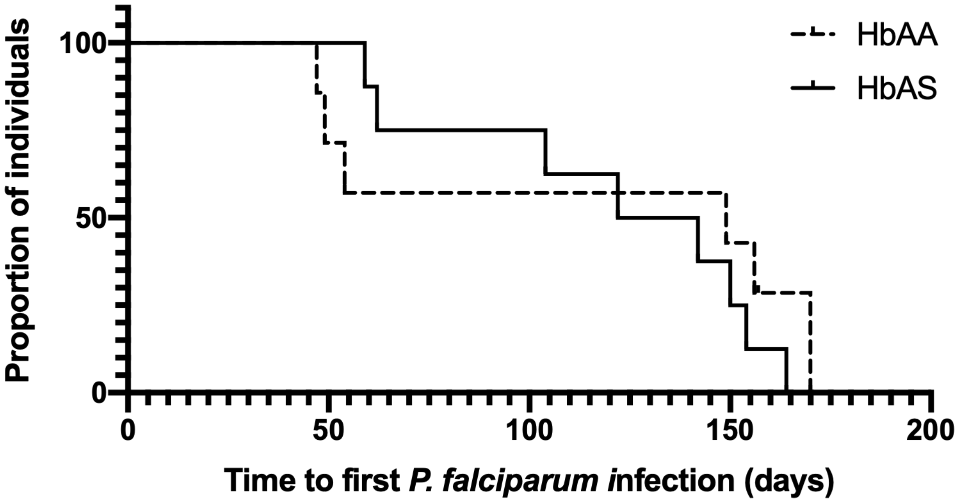

Supplementary figure 8. Kaplan-Meier curve of time to first *P. falciparum* infection, for HbAA versus HbAS individuals.

Kaplan Meier curve of the time to first *P. falciparum* infection of the malaria transmission season for HbAS (sold line) and HbAA (dotted line) individuals, presented as the days from the start of the *P. falciparum* transmission season to the first clinical malaria episode (x-axis) for the proportion of children in each group (y-axis).
